# Hybridization with Insect Cecropin A (1–8) Improve the Stability and Selectivity of Naturally Occurring Peptides

**DOI:** 10.3390/ijms21041470

**Published:** 2020-02-21

**Authors:** Yang Yang, Di Wu, Chenxi Wang, Anshan Shan, Chongpeng Bi, Yanbing Li, Wenping Gan

**Affiliations:** 1 Institute of Animal Nutrition, Northeast Agricultural University, Harbin 150030, China; yangyang_05may@163.com (Y.Y.); ohwoody@163.com (D.W.); wcx1398132690@163.com (C.W.); bnm0722@163.com (C.B.); 2 College of Animal Science and Veterinary Medicine, Heilongjiang Bayi Agricultural University, Daqing 163319, China; liyanbing929@163.com; 3 Institute of Animal Husbandry and Veterinary Medicine, Heilongjiang Academy of Land Reclamation Sciences, Harbin 150038, China; gwp30000@163.com

**Keywords:** antimicrobial peptides, hybridization, cecropin A, stability, cell selectivity, membrane disrupting mechanism

## Abstract

Antimicrobial peptides (AMPs) offer great hope and a promising opportunity to overcome the rapid development of drug-resistant pathogenic microbes. However, AMPs often lack the stability required for a successful systemic drug. Hybridizing different AMPs is a simple and effective strategy to obtain novel peptides. N-terminal fragment of cecropin A (CA (1-8)) is often used to hybridize with other AMPs to reduce cytotoxicity. However, hybridizing with CA (1-8) in improving the stability of AMPs is not clear. Therefore, a series of peptides were designed by combining with CA (1–8) and their antibacterial activity and stability in the presence of salts and human serum were evaluated. The resultant α-helical hybrid peptide CA-FO composed of CA (1-8) and the most potent region of Fowlicidin-2 (FO (1–15)) exhibited excellent antibacterial activity (2-8 μM) and cell selectivity toward bacterial over mammalian cells. Moreover, CA-FO still retained vigorous antimicrobial activity in the presence of human serum and salts at physiological concentrations. CA-FO exhibited effective antibacterial activity by increasing membrane permeability and damaging membrane integrity. In conclusion, these results indicated the success of hybridization in designing and optimizing AMPs with improved stability and selectivity and the peptide CA-FO can be further evaluated as peptide-therapy to treat bacterial infections.

## 1. Introduction

Antimicrobial resistance (AMR) has become one of the most severe threats to global public health. The declining effectiveness of traditional antibiotics has resulted in higher mortality and financial cost [1,2]. Therefore, there is an urgent need to prevent the spread and development of AMR. During the past few years, intensive researches have invested into identification of novel antibiotics. Antimicrobial peptides (AMPs) have rapidly attracted attention as potential candidate for developing novel therapeutic agents [3,4]. AMPs that serve as the chemical barrier of the innate immune system in various organisms [5,6] are broad-spectrum endogenous antibiotics effective against a broad range of invading pathogens, including Gram-positive and negative bacteria, fungi as well as viruses [7,8,9,10]. AMPs can be classified into four major groups based on their secondary structure—liner α-helical peptides, β-sheet-containing peptides, peptides involving α- and β-elements and extended peptides [11,12]. The newest research has grouped AMPs with cyclic and other complex topologies into a fifth category of “topologically complex” AMPs [13]. Despite the diversity of the length of the sequence, primary and secondary structures, most AMPs share two common characteristics which are essential for its biological activities and primary action mechanism—positive charges and hydrophobicity [14,15]. The positive charges facilitate the initial electrostatic interaction between AMPs and negatively charged lipopolysaccharides (LPS) of Gram-negative bacteria or lipoteichoic acids (LTA) of Gram-positive bacteria. After the binding between peptides and microbial membrane, the hydrophobic region of a peptide will insert into the interior and induce membrane disruption [15,16]. Membrane disruption mechanism of AMPs include barrel-stave mechanism, carpet mechanism and toroidal pore mechanism [15,17]. However, a large number of evidences has demonstrated that many AMPs exhibit antimicrobial effects without disrupting bacterial membranes but by traversing the membrane and interacting with intracellular targets such as DNA and RNA or acting as metabolism inhibitors [18,19]. Such complex and multiple mechanisms of AMPs reduce the probability of resistance development, which highlight their promise as novel antimicrobial agents.

However, naturally occurring antimicrobial peptides have serval intrinsic drawbacks that limit their development into successful therapeutic agents. These drawbacks include potential toxicity, high production costs and instability in body fluid with high salt concentration [20,21]. To overcome these problems, some modifications have been proposed, including residue substitution [22], hybridization of naturally occurring peptides, cyclization of linear peptides [23]. Hybridization could take advantage of different functional sequences, which make it an attractive approach to obtain novel AMPs [24,25]. Cecropin A, isolated from the hemolymph of *Hyalophora cecropia*, possesses potent antibacterial activity and no toxicity toward eukaryotic cells. The highly α-helical 1–8 region of cecropin A (CA (1–8)) was always used in hybridizing with others to obtain peptides with improved antimicrobial activity and cell selectivity [26,27,28,29,30]. As mentioned above, AMPs often lack the stability required for a successful drug. Until now, only a few studies have evaluated the stability of CA (1–8)-based hybrid peptides. Therefore, a series of hybrid peptides were designed by combining with CA (1–8) and their antibacterial activity and stability in the presence of salts and serum were evaluated. We speculated that an effective antimicrobial peptide with improved stability could be obtained by hybridization. Fowlicidin-2 identified in chicken is a cathelicidin peptide. Mature fowlicidin-2 of 31 amino acid residues display potent and rapid antibacterial activity against pathogens but noticeable toxic effects on mammalian cells [31]. Tritrpticin, an arginine and tryptophan-rich cathelicidin peptide, has a broad-spectrum of antimicrobial activity with low hemolytic activity [32]. A previous study has indicated that the antibacterial activities of fowlicidin-2 and tritrpticin were inhibited by the addition of salts and porcine serum [33]. In the present study, the hybrid peptides CA-FO and CA-TP were designed by combining CA (1–8) with the most potent region of Fowlicidin-2 (1–15) [33] or the antimicrobial center of tritrpticin (7–13) [34,35]. We indicated the success of hybridization in designing AMPs with increased salt resistance and cell selectivity. Consistent with the hypothesis, the novel hybrid peptide CA-FO exhibited potent antimicrobial activity even in high salt concentration. CA-FO killed bacteria through membrane disrupting mechanism, which reduced the likelihood of resistance development.

## 2. Results

### 2.1. Physicochemical Parameters of Peptides

The physicochemical parameters of the designed peptides were listed in Table 1. The measured average masses (M_av_) were confirmed using matrix-assisted laser desorption/ionization time-of-flight mass spectrometry (MALDI-TOF-MS) (Figures S1–S5). The measured M_av_ matched closely with theoretical M_av_, which proved that all the peptides had been synthesized successfully. The purity of all peptides was higher than 95%. Grand average of hydropathicity (GRAVY) value was used to predict the hydrophobicity of peptide in the present study. Greater positive GRAVY score indicates higher hydrophobicity and vice versa. Therefore, the hydrophobicity of these peptides decreased in the order of CA, CA-TP, TP, CA-FO, FO which corresponding to the declined order of GRAVY values (−0.675, −0.747, −0.829, −1.109, −1.340).

### 2.2. Secondary Structures

**Figure 1:**
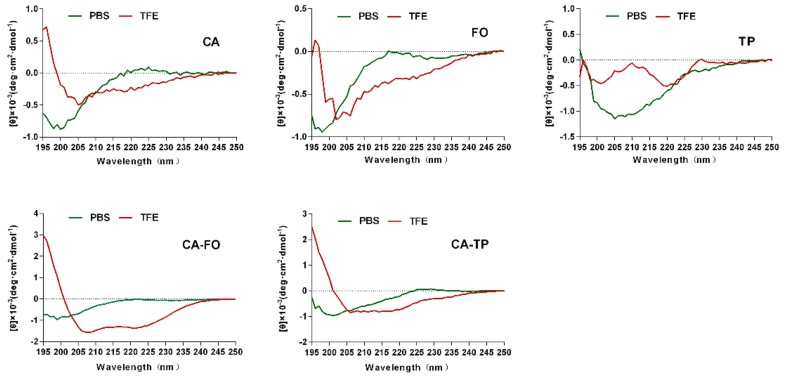
Circular dichroism (CD) spectra of all peptides. All peptides were dissolved in 10 mM phosphate-buffered saline (PBS) (pH7.4) and 50% trifluoroethanol (TFE). The mean residual ellipticity was plotted against wavelength. The values from three scans were averaged per sample and the peptide concentrations were fixed at 150 μM.

The secondary structures of the peptides were studied using circular dichroism (CD). Figure 1 showed the CD spectra of the peptides in 10 mM phosphate-buffered saline (PBS) (mimicking an aqueous environment) and 50% trifluoroethanol (TFE) (mimicking the hydrophobic environment of the microbial cell membrane). All tested peptides were characteristic of unordered structure in 10 mM PBS. In 50% TFE, CA showed a slight tendency to form helical structure compared to unordered structure of the other two parental peptides (FO and TP). Hybrid peptides CA-FO and CA-TP exhibited different degrees of α-helical conformation with minima at 208 and 222 nm.

### 2.3. Antimicrobial Activity

The minimum inhibitory concentrations (MICs) of the peptides against various Gram-positive and Gram-negative bacteria were presented in Table 2. Two hybrid peptides (CA-FO and CA-TP) showed enhanced antimicrobial activity against all tested bacterial strains with MICs range from 2 to 8 μM. CA-FO and CA-TP displayed approximately 8–64 and 16–64-fold higher antimicrobial activity than their respective parental peptides, respectively.

### 2.4. Hemolytic Activity and Cytotoxicity of the Peptides

**Figure 2:**
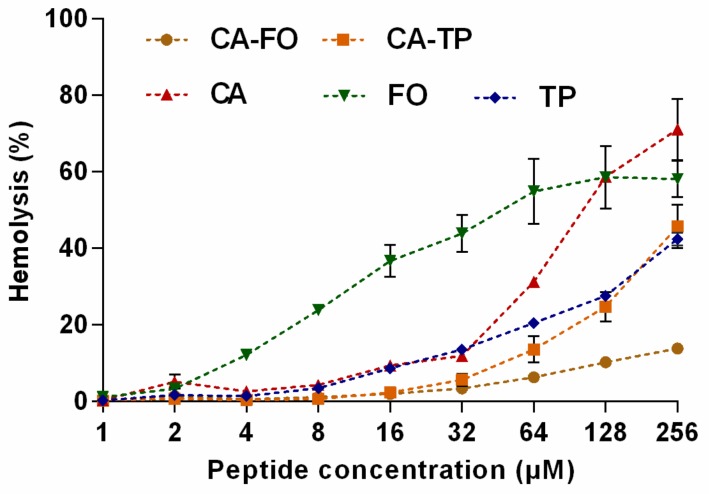
Hemolytic activity of all peptides against human erythrocytes. Human erythrocytes were treated with peptides (0.5–256 µM) at 37 °C for 1 h. Data represent average ± SEM of three independent experiments.

The hemolytic activity of the peptides was evaluated using human erythrocytes and the results were summarized in Figure 2. The hybrid peptide CA-FO demonstrated the lowest hemolytic activity among these peptides, namely, 13.83%, even at the highest concentration of 256 μM, showing an approximately 5-fold decrease compared to the hemolysis induced by CA. While the hemolytic activity of the other hybrid peptide CA-TP was not desirable at high concentration. But take their antibacterial activities into account, these two peptides hardly caused hemolysis at their MIC value. In conclusion, hybrid peptides exhibited significantly reduced hemolytic activity compared to their parental peptides.

**Figure 3:**
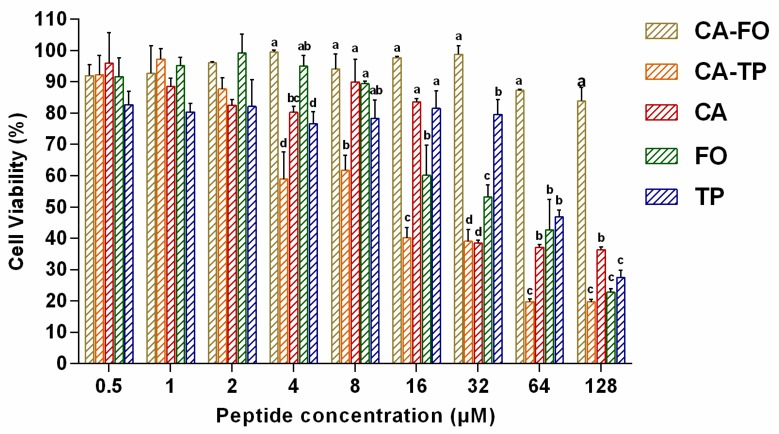
Effects of all designed peptides on RAW264.7 macrophages viability. Cells were treated with peptides (0.5–128 µM) for 24 h and viability was determined by 3-[4,5-dimethylthiozol-2-yl]-2,5-diphenyltetrazolium bromide (MTT)MTT assay. Data represent average ± SEM of three independent experiments. Significance determined using one-way ANOVA.

The effect of these peptides on cell viability was determined using 3-[4,5-dimethylthiozol-2-yl]-2,5-diphenyltetrazolium (MTT) assay. The percentage of cell viability of RAW264.7 macrophages were shown in Figure 3. At the highest concentration of 128 μM, the cell viability of CA, FO and TP were 36.36%, 22.81% and 27.49%, respectively. The hybrid peptide CA-TP almost eliminated a large proportion of living cells, with the lowest cell viability of 20% at 128 μM. However, the hybrid peptides CA-FO maintained relatively higher cell survival rates (83.88%) at 128 μM indicating the lowest toxicity among all peptides.

### 2.5. Cell Selectivity of Peptides

To evaluate the cell selectivity of all designed peptides, the therapeutic index (TI) values were calculated as HC_10_/GM. Larger TI value indicate greater cell selectivity. As shown in Table 3, CA-FO displayed the highest TI value of 41.80 indicating the greatest cell selectivity toward bacterial cells over human erythrocytes.

### 2.6. Salts and Serum Stability

The sensitivity of the peptides to salts was investigated by monitoring the changes in the MICs. As shown in Table 4, CA-FO and CA-TP retained their activities against *S. aureus* ATCC 29213 in the presence of salts. However, the antimicrobial activities of these two hybrid peptides against *E. coli* ATCC 25922 was significantly reduced by physiological salts. The addition of Ca^2+^ and the mixture of all salts completely destroyed the activities of these two peptides against *E. coli* ATCC 25922.

**Figure 4:**
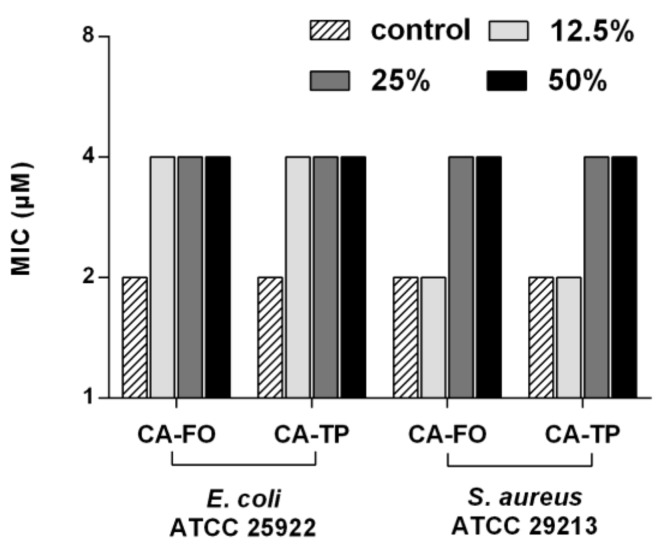
MICs of the peptides against *E. coli ATCC* 25922 and *S. aureus* ATCC29213 in the presence of human serum. Peptides were preincubated with human serum (final concentration of 50% *v*/*v*, 25% *v*/*v* and 12.5% *v*/*v*) at 37 °C for 2h before implementing MIC assay.

The stability of CA-FO and CA-TP in the presence of human serum was assessed by the change in their MICs and the result was shown in Figure 4. After incubating with 12.5%, 25% and 50% serum for 2h, CA-FO and CA-TP still retained strong antimicrobial activity against *E. coli* ATCC 25922 and *S. aureus* ATCC 29213 with MIC values of 4 μM.

### 2.7. Outer Membrane Permeabilization

**Figure 5:**
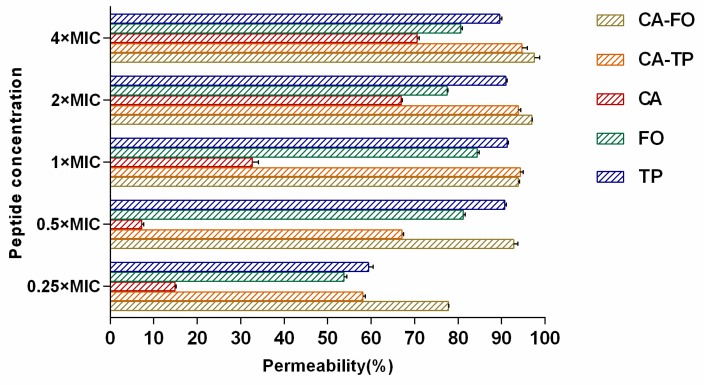
The outer membrane permeability of *E. coli* ATCC 25922 treated by different concentrations of peptides, as assessed by the fluorescence induced by hydrophobic dye 1-N-phenylnaphthylamine (NPN). Fluorescence intensity was monitored at an excitation wavelength of 350 nm and an emission wavelength of 420 nm.

1-N-phenylnaphthylamine (NPN) is a type of hydrophobic fluorescent probe that fluoresces weakly in a hydrophilic environment but generates strong fluorescence signals in a hydrophobic environment, such as the outer membrane of bacteria. Permeabilization of the outer membrane induced by AMPs creates a partitioning for NPN molecules, thereby leading to enhanced fluorescence in the cells. As shown in Figure 5, CA-FO and CA-TP increased outer membrane permeability in a dose-dependent manner. These two hybrid peptides induced more than 50% membrane permeability at their 0.25×MIC, indicating a high efficiency in membrane permeabilization.

### 2.8. Cytoplasmic Membrane Depolarization

**Figure 6:**
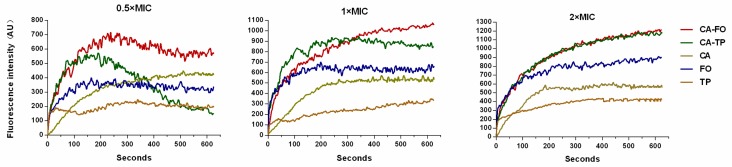
The cytoplasmic membrane potential variation of *E. coli* ATCC 25922 treated by different concentrations of peptides, as assessed by the release of the membrane potential-sensitive dye 3,3′-dipropylthiadicarbocyanine (DiSC3–5). Fluorescence intensity was monitored at an excitation wavelength of 622 nm and an emission wavelength of 670 nm as a function of time.

The ability of peptides to induce cytoplasmic membrane depolarization of *E. coli* was analyzed using voltage sensitive dye 3,3′-dipropylthiadicarbocyanine (DiSC3–5). The signal of DiSC3–5 decreases as the dye partitions to the surface of polarized cells. Upon the addition of membrane disrupting peptide, the membrane potential is rapid dissipated resulting in the release of DiSC3–5 into medium [36]. The membrane depolarization induced by the peptides was monitored over a period of 600 s. As shown in Figure 6, CA-FO caused more rapid and stronger membrane depolarization than the others. CA-FO and CA-TP showed a moderating trend after 200 s at 0.5×MIC. Of all investigated peptides, CA-FO induced the sustained increase in fluorescence intensity at 1×MIC. All tested peptides induced a higher fluorescence extend at 2×MICs compare to that induced with 1×MICs.

### 2.9. Membrane Morphological Analysis

**Figure 7:**
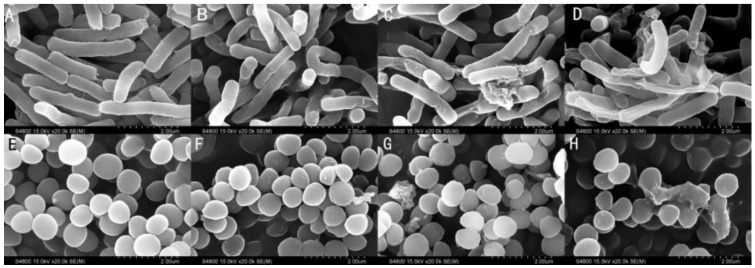
Scanning electron microscope (SEM) micrographs of *E. coli* ATCC 25922 (**A**–**D**) and *S. aureus* ATCC 29213 (**E**–**H**) cells treated with CA, CA-TP and CA-FO at their 1×MICs for 90 min. control, without peptide (**A**,**E**); CA at 1×MIC (B and F); CA-TP at 1×MIC (**C**,**G**); CA-FO (**D**,**H**) at 1×MIC.

**Figure 8:**
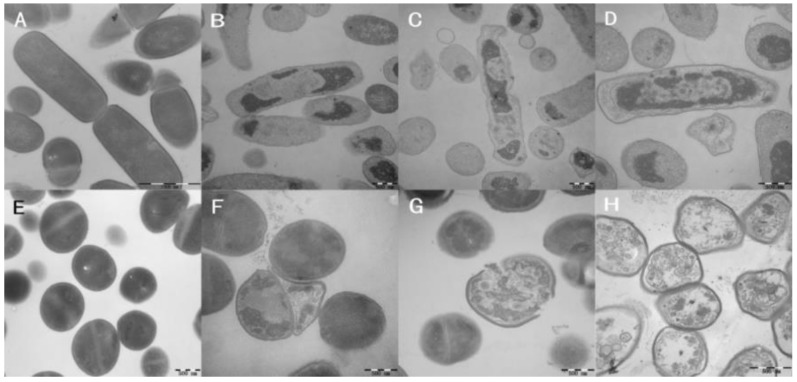
Transmission electron microscope (TEM) micrographs of *E. coli* ATCC 25922 (**A**–**D**) and *S. aureus* ATCC 29213 (**E**–**H**) cells treated with CA, CA-TP and CA-FO at their 1×MICs for 90 min. control, without peptide (**A**,**E**); CA at 1×MIC (**B**,**F**); CA-TP at 1×MIC (**C**,**G**); CA-FO (**D**,**H**) at 1×MIC.

Transmission electron microscope (TEM) and scanning electron microscope (SEM) were employed to observe the membrane integrity of the peptide-treated cells. As shown in Figure 7A (*E. coli* cells) and 7E (*S. aureus* cells), bacterial cells exhibited intact and relatively smooth cell membranes in the absence of peptides. The membrane surface of *E. coli* cells exposed to the peptides appeared to be rough and irregular exhibited atrophy. Cells treated with CA-FO exhibited the greatest degree of atrophy. *S. aureus* cells treated with CA-FO and CA-TP showed blebbier and rougher membrane surfaces (Figure 7G,H). Compared with the bright clearly continuous double membrane of untreated bacteria (Figure 8A,E), large cytoplasmic vacuoles in peptide-treated *E. coli* (Figure 8B–D) and *S. aureus* (Figure 8F–H) were observed. Simultaneously, significant breaking and fracturing of the *S. aureus* cell membrane was observed after incubation with CA-TP (Figure 8G).

## 3. Discussion

AMPs offer great hope and promising opportunity to overcome the rapid development of drug-resistant pathogenic microbes. However, the application of naturally occurring peptides is impeded by serval factors, such as high synthetic cost and limited stability [37]. When peptides are modified to evade those impediments, some parameters affecting the activity such as cation charge, hydrophobicity and secondary structure must be taken into account.

Some of peptides adopt unstructured or extended conformations in the aqueous solution. While others are conformed by intramolecular bonds [38]. Upon absorbing onto the membrane, AMPs may undergo conformational transitions to helical or β-sheet conformations that affect antimicrobial activity and specificity [33,39]. In the present study, hybrid peptides CA-FO and CA-TP were unordered in PBS and exhibited the same α-helical structure as the parental peptide CA in the presence of 50% TFE. The characteristic negative ellipticity of α-helical conformation became more obvious in hybrid peptides. The high helical propensities of CA-FO and CA-TP were caused by the increase of chain length. Generally, the improvement of helical propensity with the increase of chain length is due to the enhancement of hydrogen-bonding interaction along the helical backbone, which stabilizes the helical structure [40,41,42]. Higher α-helical propensity leads to greater affinity between peptides and microbial membranes, resulting in better membrane permeabilization in bacteria [43]. In present study, hybrid peptides, CA-TP and CA-FO, with higher helical contents possessed remarkable antimicrobial activity than their parental peptides (Table 2). Cationic charge also had an initial influence on antimicrobial activity of the peptides. Enhancement of cationic charge facilitates the electrostatic interaction of peptides with the negatively charged bacterial membrane [44]. CA-FO and CA-TP with higher cationic charge than their parental peptides exhibited higher antimicrobial activity. It is widely accepted that after binding to a microbial membrane, the hydrophobic region of a peptide provides a lipophilic anchor and destroys membrane integrity and eventually causes cell death [45]. However, antimicrobial activity is not positively correlated with antibacterial activity. There is a threshold value for optimized hydrophobicity over which increase in hydrophobicity lead to loss of antimicrobial activity [46]. Therefore, CA with the highest hydrophobicity had no bactericidal effect.

Hemolysis and cytotoxicity are important indicators of the toxic effect of the peptides to mammalian cells. Peptides with higher hydrophobicity led to deeper insertion into microbial membranes but at the same time, they tended to penetrate deeper into the hydrophobic interiors of human cells, leading to increases in both antimicrobial activity and cytotoxic activity [47]. At the MIC levels both CA-FO and CA-TP had lower hemolytic activity than the parental peptide CA, presumably due to the rational hydrophobicity of these peptides. Composition of amino acid is another factor affecting the cytotoxicity. Although FO displayed the minimal hydrophobicity, it possessed high toxic toward mammalian cells because of the high proportion (20%) of phenylalanine (Phe). Previously studies have shown that AMPs with high mount of Phe are often accompanied by high cytotoxicity [48,49].

The significant reduction of antimicrobial potency under certain biological conditions has impeded the further development of AMPs into systemic drug. This inhibitory effect exerted by biological fluids is resulted from serval factors, such as the presence of high concentrations of salt and serum protein [37]. Primarily, the presence of salts with high concentrations may disturb the electrostatic interaction between the peptides and bacterial membrane [50]. In the present study, the sensitivity of the peptide to salts at physiological concentrations was investigated by monitoring the changes in the MIC values. As shown in Table 4, hybrid peptide CA-FO and CA-TP retained vigorous activities against *S. aureus* ATCC 29213 in the presence of salts. High net charge (+13) presented by CA-FO was enough to neutralize the charge screening effect induced by the addition of salts, resulting in maintained antibacterial activity. The result agrees with previous reports, peptides with higher net charge were less sensitive to salts [33,51]. Unlike CA-FO, CA-TP presented lower net charge of +7 but higher hydrophobicity. The addition of salts hampered the peptide-bacterium electrostatic interactions, which decreased the effective concentration of peptide on bacterial membrane but higher hydrophobicity of CA-TP ensured the deeper membrane insertion, which ensured the disruption of microbial membrane. However, the activities of these two hybrid peptides against *E. coli* ATCC 25922 were more susceptible to the addition of salts. This phenomenon could be explained by the different architecture in the enveloped of Gram-negative and Gram-positive bacteria. Gram-negative bacteria have an additional outer membrane coated with lipopolysaccharide (LPS). The divalent cations (Ca^2+^ and Mg^2+^) neutralize the repulsive force generated by accumulation of negative charged LPS which maintain the integrity and stability of the outer membrane [15,52]. Therefore, the antibacterial activity of the peptide against Gram-negative is more easily affected by the disruption of electrostatic adsorption due to low permeability of membrane. As mentioned above, in addition to salts, serum protein such as albumin can bind to peptides and consequently reduce the effective concentration of peptides. Furthermore, the binding of serum protein to the bacterial surface mask the binding site on bacterial membrane and reduce the antibacterial activity of peptides [53]. In the present, the addition of human serum only partially inhibited the antibacterial activity of CA-TP and CA-FO, these two hybrid peptides retained desirable antibacterial activity with the MIC values of 4 μM (Figure 4). As indicated by Deslouches [54], there is an optimal balance between free and protein-bound peptide molecules. As more free peptides become associated with their targeted membrane, the equilibrium would shift toward the progressive release of more peptide resulting in the retention of antimicrobial activity.

Most cationic antimicrobial peptides exhibit their antibacterial activity by destroying the cell membranes, through mechanisms ranging from permeabilization to depolarization and transient gaps [55,56]. In present study, outer membrane permeability and cytoplasmic membrane potential assays were determined to investigate the interaction between the hybrid peptides and the cell membrane. The enhanced fluorescence of NPN molecule showed clear evidence of the dose-dependent interaction of hybrid peptides with the bacterial outer membrane (Figure 5). Subsequently, the cytoplasmic membrane potential assay indicated that CA-FO possessed the ability to damage the bacterial cytoplasmic membrane (Figure 6). As expected, the hybrid peptides destroyed the cell membrane in a dose and time-dependent manner. Membrane destruction leading to loss of barrier function would result in the leakage of cell cytoplasmic content and cell death. SEM and TEM studies further confirmed that the peptides had dramatic impact on membranes integrity, providing morphological evidence of the membrane-disrupted activity of the peptides (Figs. 7 and 8).

## 4. Materials and Methods

### 4.1. Bacterial Strains and Mammalian Cells

The bacterial strains *E. coli* ATCC 25922, *S. typhimurium* C 7731, *S. typhimurium* ATCC 14028, *P. aeruginosa* ATCC 27853, *S. pullorum* C 7913, *S. aureus* ATCC 29213, *S. epidermidis* ATCC 12228 and *E. faecalis* ATCC 29212 were obtained from the College of Veterinary Medicine, Northeast Agricultural University (Harbin, China). *E. coli* UB1005 was provided by the State Key Laboratory of Microbial Technology, Shandong University (Jinan, China). RAW264.7 was purchased from the cell bank of the Chinese Academy of Science, SIBS (Shanghai, China).

### 4.2. Peptide Synthesis and Sequence Analysis

The peptides designed in this study were synthesized by GL Biochem (Shanghai, China). The precise molecular mass was confirmed using matrix-assisted laser desorption/ionization time-of-flight mass spectrometry (MALDI-TOF-MS) (MALDI-7090, Shimadzu Kratos, Manchester, UK). The purity of designed peptides was determined by reverse-phase high performance liquid chromatography (RP-HPLC) (LC3000, Beijing, China) on a Gemini-NX C18 column (250 × 4.60 mm, with 5 μm internal particles) and the detection wavelength was 220 nm. All these peptides had a purity higher than 95%.

Primary peptide sequence analyses were performed using the bioinformatics programs ProtParam (ExPASy Proteomics Server: http://web.expasy.org/protparam/). Charge and grand average of hydropathicity (GRAVY) were calculated online using HeliQuest (http://heliquest.ipmc.cnrs.fr/cgi-bin/ComputParamsV2.py) and ExPASy (https://web.expasy.org/protparam/).

### 4.3. CD Spectroscopy

CD spectra of the peptides in different environments were obtained at 25 °C on a J-820 spectropolarimeter (Jasco, Tokyo, Japan) using a 1-mm-path-length quartz cell. Peptides were scanned at a concentration of 150 μM in 10 mM PBS (pH 7.4) and 50% TFE (Amresco, Solon, OH, USA). CD spectra were recorded at a scanning speed of 10 nm/min in the 195 to 250 nm wavelength range and each spectrum was the average of three scans. The mean residue ellipticity (θ_M_, deg•cm^2^•dmol^−1^) was obtained by the following formula:

θ_M_ = (θ_obs_·1000)/(c·l·n) (1)

where θ_obs_ is the observed ellipticity (mdeg), c is the concentration (mM) of peptide solution, l is the path length (mm) and n is the number of residues.

### 4.4. Antimicrobial Assays

Minimum inhibitory concentrations (MICs) were determined as previously described [57]. The bacteria used to determine the MIC were first cultured in Mueller-Hilton broth (MHB) (AoBoX, Beijing, China) overnight at 37 °C and transferred into a fresh and sterile MHB and grown to an OD600 of 0.4. The bacteria then adjusted to a cell density of 5×10^5^ CFU mL^−1^ in MHB. Peptides were serially diluted in 0.01% acetic acid and 0.2% bovine serum albumin (BSA) (Sigma-Aldrich, Shanghai, China) in a volume of 50 μL per well to achieve final concentration ranging from 1 to 128 μM in sterile 96-well plates. 50 μL of prepared bacterial suspension was added to each well. The mixtures were incubated at 37 °C for 18–24 h. The MICs were calculated as the lowest concentration of the peptide that prevented visible turbidity. The tests were performed in triplicate using three replicates for each experiment.

### 4.5. Measurement of Hemolytic Activity

Hemolytic activities of the peptides were determined according to the method described previously [58]. Fresh heparinized human whole blood from healthy donor (Yang Yang, Harbin, China) was centrifuged at 1000 g for 5 min at 4 °C. The obtained erythrocytes were washed three times and resuspended in PBS. Then, 50 μL of erythrocyte solution was incubated with 50 μL peptides at different concentrations at 37 °C for 1 h. Erythrocytes in either PBS or 0.1% Triton X-100 (Sigma-Aldrich, Shanghai, China) were used as negative and positive controls, respectively. After centrifugation at 1000 g for 5 min at 4 °C, the supernatant was transferred into a new 96-well plate and the extent of hemolysis was monitored using a multimode microplate reader (Infinite M200 Pro, Tecan, Switzerland) at 576 nm. The percentage of hemolysis was calculated according to the following formula:

hemolysis (%) = [(A-A_0_)/(A_t_-A_0_)]×100 (2)

where A is the absorbance of the peptide sample, A_0_ and A_t_ represent 0% and 100% hemolysis determined in 10 mM PBS and 0.1% Triton X-100, respectively. Results were expressed as average values from three independent experiments and each experiment was performed in triplicate.

### 4.6. Cytotoxicity Assays

The cytotoxicity of designed peptides was determined using MTT assays, as previously described [59]. Viable cells metabolize MTT into water-insoluble formazan crystals. The formation of formazan is proportional to the number of functional mitochondria in living cells [60]. RAW267.4 murine macrophages in RPMI-1640 medium (HyClone, GE Healthcare Life Sciences, USA) (supplemented with 10% fetal bovine serum) were seeded in 96-well plate at a concentration of 1~2×10^5^ cells/well, followed by an overnight incubation at 37 °C in 5% CO_2_. Peptides were added to cell cultures at various concentrations ranging from 0.5 to 128 µM. Untreated cells and wells without cells served as controls and blanks, respectively. The mixtures were further incubated for 24 h. Subsequently, 50 μL of MTT (Sigma-Aldrich, Shanghai, China) (0.5 mg mL^−1^) was added to each well and the plates were incubated for 4 h at 37 °C. After incubation, 96-well plates were centrifuged at 1000 g for 5 min and the supernatants were discarded and 150 μL of dimethyl sulfoxide (DMSO) (Sigma-Aldrich, Shanghai, China) was added to dissolve the formazan crystals. Finally, the absorbance at 570 nm was measured with a multi-mode microplate reader (Infinite M200 Pro, Tecan, Switzerland). A total of three replicates were conducted for each concentration.

### 4.7. Calculation of the Therapeutic Index

The therapeutic index is a quantitative measurement of the cell specificity of antimicrobial peptides. It is calculated as HC_10_/GM. HC_10_ is the minimal inhibitory concentration that induced 10% hemolysis of human red blood cells. GM is the geometric mean of the MIC values of a peptide against all the tested bacterial strains. When no detectable antimicrobial activity was observed at 128 μM, a value of 256 μM was used for calculation of the GM value. The larger the therapeutic index (TI), the better cell selectivity the peptide has.

### 4.8. Salt and Serum Stability

*E. coli* ATCC 25922 and *S. aureus* ATCC 29213 cells were diluted in the presence of different concentrations of salt (150 mM NaCl, 4.5 mM KCl, 6 μM NH_4_Cl, 8 μM ZnCl_2_, 1 mM MgCl_2_ 2 mM CaCl_2_ and 4 μM FeCl_3_) [61]. Subsequent steps were consistent with the MICs determination method.

Serum stability was determined according to the method previously described [62]. Human serum was added in the peptide solution with the volume ratio of 1:1 in which the final concentration of 50% *v*/*v* of serum was yielded. Serum-peptide mixtures with the final concentration of 25% and 12.5% *v*/*v* serum were also prepared. Subsequently, the mixture was incubated at 37 °C for 2h. The antimicrobial activities of serum-preincubated peptides were determined according the MIC assay described above.

### 4.9. Outer Membrane Permeability Assay

*E. coli* ATCC 25922 was grown overnight in MHB at 37 °C and transferred into a fresh and sterile MHB and grown to mid-log phase. Cells were washed and resuspended in 5 mM HEPES buffer (containing 5 mM glucose, pH 7.4) to an OD600 of 0.2 in the presence of 10 μM NPN (Sigma-Aldrich, MO, USA). The background fluorescence was recorded using excitation and emission wavelengths of 350 nm and 420 nm, respectively, with an F-4500 fluorescence spectrophotometer (Hitachi, Japan). Different concentrations of peptides were added to the cells and the fluorescence was recorded with time until no further increase was observed. The values were converted to %NPN uptake using the following equation:

%NPN = [(F_obs_-F_0_)/(F_100_-F_0_)] (3)

where F_obs_ is the observed fluorescence intensity after addition of peptide, F_0_ is the basal fluorescence intensity of NPN with *E. coli* cells in the absence of peptides and F_100_ is the fluorescence intensity after addition of 10 mg mL^−1^ polymyxin B (Sigma-Aldrich, Shanghai, China), which was used as a positive control in this experiment.

### 4.10. Cytoplasmic Membrane Depolarization Assay

DiSC3–5 was used for the determination of cytoplasmic membrane potential. *E. coli* ATCC 25922 was grown overnight in MHB broth and then centrifuged at 1000 g for 10 min. Cell pellets were then washed three times with 10 mM phosphate buffer at pH 7.4 and resuspended to an OD600 of 0.05 with 5 mM HEPES buffer (pH 7.4, containing 20 mM glucose). Following the addition of 0.4 μM DiSC3–5 (Sigma-Aldrich, Shanghai, China), samples were incubated for 90 min in the dark to obtain a stable reduction of fluorescence. KCl (4 M) was added to the cell suspension to achieve a final concentration of 100 mM. 2 mL of cell suspension was added to a 1 cm quartz cuvette and mixed with peptides. Fluorescence was measured with an F-4500 fluorescence spectrophotometer (Hitachi, Japan) at excitation/emission wavelengths of 622/670 nm, respectively.

### 4.11. SEM and TEM Characterization

SEM and TEM were carried out as previously described [59]. *E. coli* ATCC 25922 and *S. aureus* ATCC 29213 were grown overnight at 37 °C and transferred into a fresh and sterile MHB and grown to an OD600 of 0.4. Cells were harvested by centrifugation at 1000 g for 10 min at 4 °C, washed three times with PBS and resuspended to an OD600 of 0.2. *E. coli* ATCC 25922 and *S. aureus* ATCC 29213 were incubated with different peptides at their 1×MICs. After 90 min incubation, the cells were fixed overnight with 2.5% glutaraldehyde at 4 °C. Samples were dehydrated in an ascending ethanol series. The specimens were then dried and coated with gold and examined using a HITACHI S-4800 SEM (Hitachi, Japan).

Microbial samples were initially prepared as described above for FE-SEM analysis. After pre-fixation with 2.5% glutaraldehyde overnight, cell pellets were washed 3 times with PBS and post-fixed with 2% osmium tetroxide in PBS for 70 min. Samples were washed twice with PBS, followed by dehydration with a graded ethanol series and immersed in pure epoxy resin in a constant-temperature incubator overnight. Finally, specimens were sectioned using an ultramicrotome, stained with uranyl acetate and lead citrate and observed using a HITACHI H-7650 TEM (Hitachi, Japan).

### 4.12. Statistical Analysis

For statistical evaluation, data were analyzed using the Statistical Product and Service Solutions software (SPSS Inc., Chicago, IL, USA) by one-way ANOVA and the values were presented as mean ± standard error (SE). HC10 values were calculated by using probit regression in SPSS 19.

## Figures and Tables

**Table 1:** Amino acid sequences and key physicochemical parameters of the peptides.

Peptide	Sequence	Theoretical M_av_ (u)	Measured M_av_ (u) ^1^	Purity ^2^ (%)	Charge	GRAVY ^3^
CA-FO	KWKLFKKIRFGRFLRKIRRFRPK-NH_2_	3104.88	3104.24	95.72%	+13	−1.109
CA-TP	KWKLFKKIWWPFLRR-NH_2_	2131.66	2132.61	97.42%	+7	−0.747
CA	KWKLFKKI-NH_2_	1089.42	1089.89	95.74%	+5	−0.675
FO	RFGRFLRKIRRFRPK-NH_2_	2032.49	2032.59	99.25%	+9	−1.340
TP	WWPFLRR-NH_2_	1059.27	1059.79	98.33%	+3	−0.829

^1^ Molecular average mass (Mav) was measured by matrix-assisted laser desorption/ionization time-of-flight mass spectrometry (MALDI-TOF-MS). ^2^ Purity was determined by reverse-phase high performance liquid chromatography (RP-HPLC). ^3^ Grand average of hydropathicity (GRAVY). Higher positive score indicates greater hydrophobicity and *vice versa*.

**Table 2:** Minimum inhibitory concentrations (MICs) of all peptides against Gram-negative and Gram-positive bacteria.

MIC (µM) ^1^	CA	FO	TP	CA-FO	CA-TP
Gram-negative bacteria					
*E. coli* ATCC25922	64	32	128	2	2
*E. coli* UB 1005	>128	16	128	2	4
*S. typhimurium* C7731	>128	32	64	4	2
*S. typhimurium* ATCC14028	>128	32	32	4	4
*P. aeruginosa* ATCC27853	>128	32	64	4	4
*S. pullorum* C7913	>128	16	64	8	4
Gram-positive bacteria					
*S. aureus* ATCC29213	128	64	64	2	2
*S. epidermidis* ATCC12228	>128	16	32	4	2
*S. faecalis* ATCC29212	>128	16	>128	4	2

^1^ The minimum inhibitory concentrations (MICs) were determined as the lowest concentration of peptide that inhibited bacterial growth.

**Table 3:** Biocompatibility of the Engineered Peptides.

Peptide	GM (μM) ^1^	HC_10_^2^	Therapeutic Index (TI) ^3^
CA	203.19	15.54	0.08
FO	25.40	2.78	0.11
TP	74.66	25.06	0.34
CA-FO	3.43	143.34	41.79
CA-TP	2.72	49.66	18.26

^1^ GM, geometric mean of the MIC values. When no detectable antimicrobial activity was observed at 128 μM, a value of 256 μM was used for calculation of the GM value. ^2^ HC_10_ is the minimal inhibitory concentration that induced 10% hemolysis of human red blood cells. ^3^ Therapeutic index (TI) is calculated as HC_10_/GM. Larger values indicate greater cell selectivity.

**Table 4:** Minimum inhibitory concentrations (MICs) of parental and hybrid peptides in the presence of physiological concentrations of different salts.

	NaCl ^1^	KCl ^1^	NH_4_Cl ^1^	MgCl_2_^1^	ZnCl_2_^1^	CaCl_2_^1^	FeCl_3_^1^	Mix ^2^	Control ^1^
*E. coli* ATCC 25922									
CA-FO	32	128	16	8	16	>128	8	>128	2
CA-TP	4	4	1	2	2	>128	4	>128	2
CA	64	>128	>128	128	>128	>128	>128	>128	64
FO	32	32	32	16	64	>128	>128	>128	32
TP	128	64	128	64	128	64	128	128	64
*S. aureus* ATCC 29213									
CA-FO	4	4	2	2	2	1	2	4	2
CA-TP	2	2	2	2	2	0.25	2	4	2
CA	128	128	>128	64	64	64	64	64	128
FO	32	32	32	16	64	64	64	64	64
TP	128	64	128	64	128	64	128	128	64

^1^ The final concentration of NaCl, KCl, NH_4_Cl, MgCl_2_, ZnCl_2_, CaCl_2_ and FeCl_3_ were 150 mM, 4.5 mM, 6 μM, 1 mM, 2 mM, 8 μM and 4 μM, respectively and the control MIC values were determined in the absence of these physiological salts. ^2^ The medium contained all kind of salts in physiological concentrations.

## Supplementary Materials

The following are available online at https://www.mdpi.com/1422-0067/21/4/1470/s1.

## Abbreviations

AMR	Antimicrobial resistance
AMPs	Antimicrobial peptides
LPS	Lipopolysaccharides
LTA	Lipoteichoic acids
MALDI-TOF-MS	Matrix-assisted laser desorption/ionization time-of-flight mass spectrometry
GRAVY	Grand average of hydropathicity
CD	Circular dichroism
PBS	Phosphate-buffered saline
TFE	Trifluoroethanol
MICs	The minimum inhibitory concentrations
MTT	3-[4,5-dimethylthiozol-2-yl]-2,5-diphenyltetrazolium bromide
TI	Therapeutic index
NPN	1-N-phenylnaphthylamine
DiSC3–5	3,3′-dipropylthiadicarbocyanine
RP-HPLC	Reverse-phase high performance liquid chromatography
