# Supplementary material for: Hybridization with Insect Cecropin A (1–8) Improve the Stability and Selectivity of Naturally Occurring Peptides
Source: Int J Mol Sci. 2020 Feb 21;21(4):1470. doi: 10.3390/ijms21041470 (PMC7073140; doi:10.3390/ijms21041470)
Supplement: Supplementary file 1 [file ijms-21-01470-s001.zip › supporting information.docx]

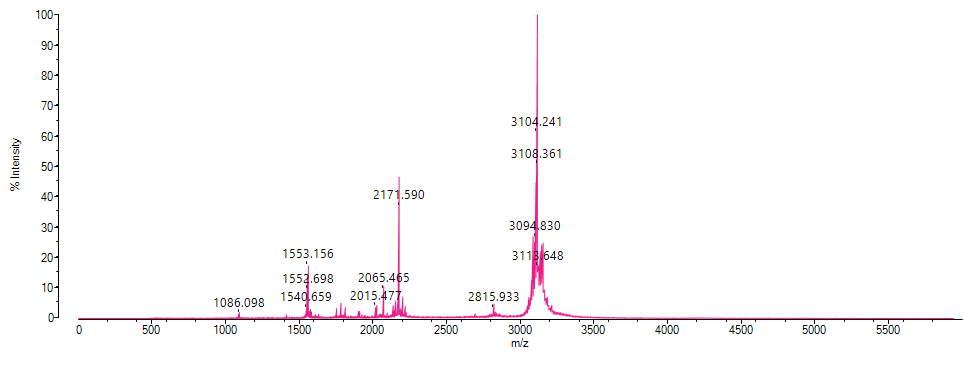
Figure S1. Mass spectrum analysis of CA-FO


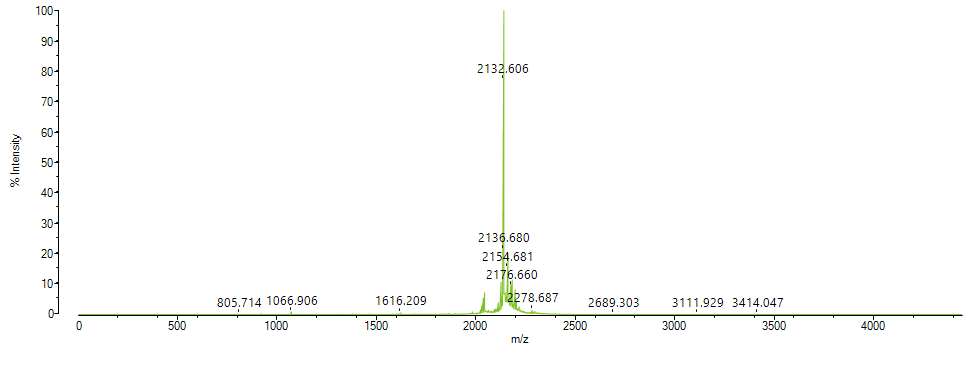
 Figure S2. Mass spectrum analysis of CA-TP


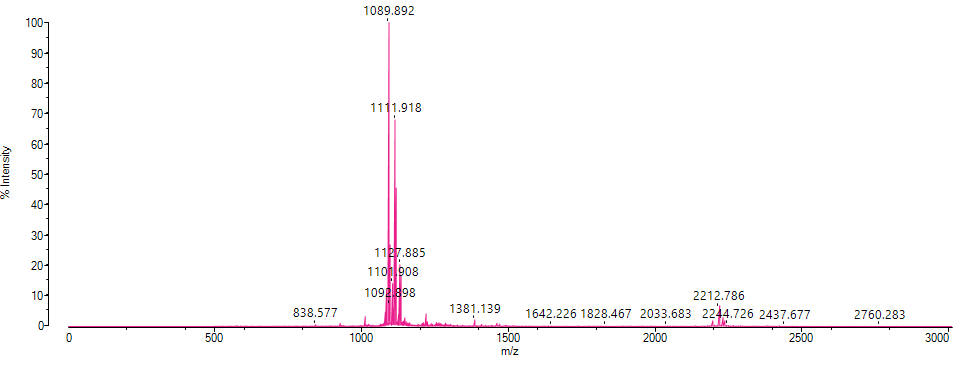


Figure S3. Mass spectrum analysis of CA


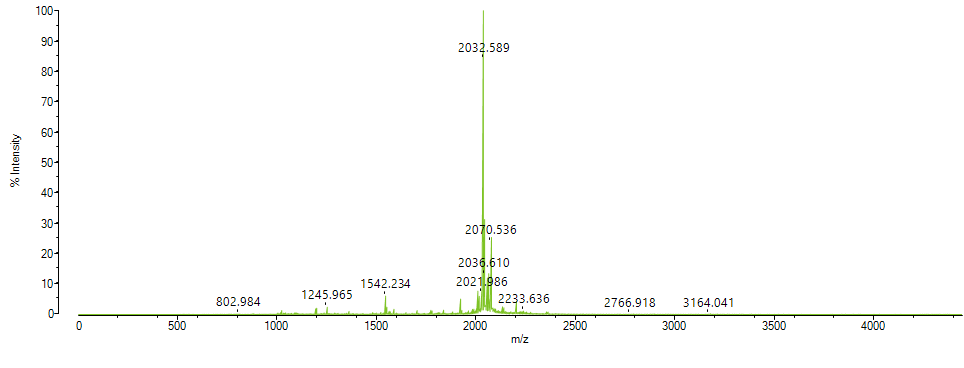
 Figure S4. Mass spectrum analysis of FO


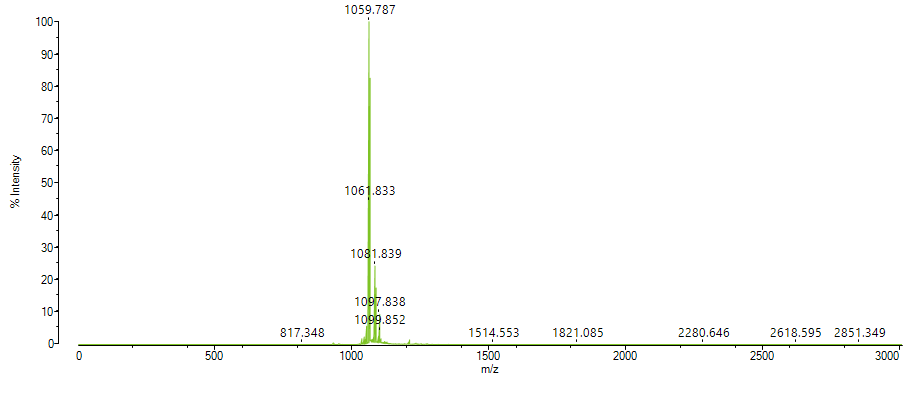


Figure S5. Mass spectrum analysis of TP
